# Supplementary material for: ABO blood group and COVID-19 severity: Associations with endothelial and adipocyte activation in critically ill patients
Source: PLoS One. 2025 Apr 2;20(4):e0320251. doi: 10.1371/journal.pone.0320251 (PMC11964209; doi:10.1371/journal.pone.0320251)
Supplement: S1 Table — (DOCX) [file pone.0320251.s001.docx]

**S1 Table.** Clinical laboratory measures [taken upon study enrollment] for COVID-19 patients admitted to VGH ICU between March 30 2020 and March 31 2021 based on ABO blood group.

|  | **A&AB**  (n=49) | **B&O**  (n=79) | **P-values^a^** |
| --- | --- | --- | --- |
| **Clinical laboratory results (reference range, male/female)** | | | |
| *Complete blood count, median [IQR]* | | | |
| WBC count, x10^9^/L (4-11) | 10.7 [7.55, 13.5] | 9.9 [6.3, 12.7] | 0.46 |
| PMN count, x10^9^/L (2-7) | 8.3 [6.0, 11.4] | 8.1 [5.7, 11.0] | 0.72 |
| Lymphocytes, x10^9^/L (1.2-4) | 0.7 [0.5, 1.0] | 0.8 [0.6, 1.0] | 0.46 |
| Hemoglobin, g/L (135-170/120-155) | 123 [109, 130] | 120 [99, 132] | 0.66 |
| Platelets, x10^9^/L (150-400) | 238 [188, 312] | 242 [168, 313] | 0.95 |
| *Coagulation, median [IQR]* | | | |
| PTT, s, median [IQR] (25-38) | 32 [28, 38] | 31 [28, 35] | 0.32 |
| INR, median [IQR] (0.9-1.2) | 1.1 [1.08, 1.2] | 1.1 [1.1, 1.2] | 0.66 |
| D-dimer, ug/L (<500) | 1031 [760, 1823] | 1497 [798, 6244] | **0.033** |
| *Liver & kidney function, median [IQR]* | | | |
| Albumin, g/L (34-50) | 26 [22, 31] | 25 [21, 29] | 0.18 |
| AST, U/L (10-38) | 61 [37, 92] | 46 [34, 106] | 0.45 |
| ALT, U/L (10-55) | 59 [42, 96] | 50 [31, 109] | 0.41 |
| Bilirubin, μmol/L (<20) | 7 [6, 14] | 8.5 [6.0, 15] | 0.52 |
| LDH, U/L (90-240) | 400 [303, 504] | 389 [284, 506] | 0.99 |
| Creatinine, μmol/L (60-115/40-95) | 94 [78, 137] | 85 [68, 121] | 0.30 |
| *Inflammation, median [IQR]* | | | |
| CRP, mg/L (<3.1) | 85 [49, 118] | 78 [31.2, 119] | 0.35 |
| Ferritin, ug/L (15-370/15-225) | 983 [491, 1531] | 1017 [481, 2165] | 0.58 |
| **^a^** Pair-wise comparisons were conducted using a Mann Whitney U Test (continuous variables) | | | |
