# Supplementary material for: ABO blood group and COVID-19 severity: Associations with endothelial and adipocyte activation in critically ill patients
Source: PLoS One. 2025 Apr 2;20(4):e0320251. doi: 10.1371/journal.pone.0320251 (PMC11964209; doi:10.1371/journal.pone.0320251)
Supplement: S2 Table — For graphical representation see Figs 1–3. (DOCX) [file pone.0320251.s002.docx]

**S2 Table.** Research laboratory measures [taken upon study enrollment] for COVID-19 patients admitted to VGH ICU between March 30 2020 and March 31 2021 based on ABO blood group. For graphical representation see Figure 1-3.

|  | **A&AB**  (n=49) | **B&O**  (n=79) | **P-values** ^a^ |
| --- | --- | --- | --- |
| **Research biomarker results** | | | |
| *Serum inflammatory markers, median [IQR]* | | | |
| IL-6, pg/mL | 42.0 [16.7, 124] | 28.3 [12.6, 97.1] | 0.52 |
| IL-1b, pg/mL | 0.148 [0.068, 0.297] | 0.130 [0.078, 0.269] | 0.77 |
| IL-10, pg/mL | 11.8 [5.21, 26.3] | 13.6 [6.05, 25.1] | 0.99 |
| TNFa, pg/mL | 8.51 [6.73, 11.8] | 8.21 [5.96, 12.0] | 0.74 |
| sIL-6R, ng/mL | 55.8 [38.1, 72.4] | 53.2 [37.9, 68.7] | 0.47 |
| sgp130, ng/mL | 240 [211, 273] | 232 [189, 295] | 0.97 |
| *Plasma endothelial markers, median [IQR]* | | | |
| Thrombomodulin (ng/mL) | 6.61 [4.34, 10.9] | 6.23 [4.50, 10.4] | 0.73 |
| vWF (parts/10,000) | 1.52 x10^4^ [1.02, 1.41 x10^4^] | 1.67 x10^4^ [0.94, 2.37 x10^4^] | 0.86 |
| ADAMTS13 (ng/mL) | 330 [267, 408] | 355 [278, 426] | 0.31 |
| sP-Selectin (ng/mL) | 49.5 [33.3, 71.3] | 53.6 [35.8, 76.9] | 0.26 |
| Factor IX (parts/10,000) | 1.00 x10^4^ [0.78, 1.21 x10^4^] | 8.92 x10^3^ [7.66, 11.7 x10^3^] | 0.48 |
| Protein C (parts/10,000) | 8.10 x10^3^ [6.44, 11.3 x10^3^] | 7.99 x10^3^ [6.47, 10.3 x10^3^] | 0.89 |
| Protein S (parts/10,000) | 7.31 x10^3^ [5.78, 8.36x10^3^] | 6.96 x10^3^ [5.89, 8.26 x10^3^] | 0.90 |
| *Plasma adipokine markers, median [IQR]* | | | |
| Adiponectin (pg/mL) | 4.82 x10^8^ [1.96, 7.92 x10^8^] | 3.13 x10^8^ [1.07, 5.95 x10^8^] | 0.066 |
| Adipsin (pg/mL) | 16.3 x10^6^ [4.2, 38.5 x10^6^] | 9.61 x10^6^ [3.0, 20.8 x10^6^] | **0.048** |
| Resistin (pg/mL) | 4.00 x10^4^ [2.56, 7.00 x10^4^] | 3.95 x10^4^ [2.40, 6.15 x10^4^] | 0.49 |
| Lipocalin-2 (pg/mL) | 2.84 x10^5^ [1.68, 5.01 x10^5^] | 3.00 x10^5^ [1.78, 5.67 x10^5^] | 0.93 |
| PAI-1 (total) (pg/mL) | 3.57 x10^4^ [2.49, 5.00 x10^4^] | 3.11 x10^4^ [1.97, 3.91 x10^4^] | 0.13 |
| **^a^** Pair-wise comparisons were conducted using a Mann Whitney U Test (continuous variables) | | | |
