# Supplementary material for: ABO blood group and COVID-19 severity: Associations with endothelial and adipocyte activation in critically ill patients
Source: PLoS One. 2025 Apr 2;20(4):e0320251. doi: 10.1371/journal.pone.0320251 (PMC11964209; doi:10.1371/journal.pone.0320251)
Supplement: Attachment — Submitted filename: Comments_Reviewed.pdf [file pone.0320251.s004.pdf]

Association of ABO Blood Group with Endothelial and Adipocyte Activation in COVID-19

Introduction:

- ✓ Role of markers of endothelial injury and adipocyte activation has not been introduced. These are the primary parameters of study that should be highlighted thoroughly.
- ✓ It looks like a discussion, moreover severity, risk factors, markers should by emphasized.

Methods:

- ✓ "as previously described." Should be removed from the line no. 167.
- ✓ Inclusion and exclusion criteria not clear.
- ✓ Time period of patient recruited is vague. Some where its written from march 2020, to march 2024 and in tables its upto march 2021.
- ✓ What was the criteria used for severity?

Results:

- ✓ Exact p value should be written.
- ✓ Comparison of the ABO blood groups with severity should be highlighted before their association with the clinical factors, even though author compared in previous studies but this should be included in supplementary.
- ✓ Steroid, antiviral, immunomodulatory drugs, anti-inflammatory used for no. of days should be highlighted and change in the levels of markers should be highlighted as it is a cohort.
- ✓ Is there any effect of drugs used on adipocyte modification and endothelial injury?
- ✓ Except from the markers do author seen any changes in the endothelium or epithelium?
- ✓ Levels of the sIL-6R, sgp130, others markers should be compared with the severity and clinical parameters especially comorbid and drugs. These are compared using the regression model so p value should be highlighted in the figures.

Discussion:

Justification of findings should be emphasized with proper refrences.
