# Supplementary material for: ABO blood group and COVID-19 severity: Associations with endothelial and adipocyte activation in critically ill patients
Source: PLoS One. 2025 Apr 2;20(4):e0320251. doi: 10.1371/journal.pone.0320251 (PMC11964209; doi:10.1371/journal.pone.0320251)
Supplement: Attachment — Submitted filename: Review of manuscript.docx [file pone.0320251.s003.docx]

**Review of the Manuscript Title**

The manuscript title should capture the study’s focus on ABO blood group associations with COVID-19 severity and relevant biological mechanisms. A well-crafted title not only reflects the key findings but also attracts readers’ attention.

1. **Incorporate Key Study Components (ABO Blood Group, COVID-19 Severity, Biological Mechanisms):**
   - **Current Title (assumed):** "Association of ABO Blood Group with Endothelial and Adipocyte Activation in COVID-19.". A more descriptive title incorporating "COVID-19 severity" and "critically ill patients" makes it clear that the study is focused on clinical outcomes and specific biological mechanisms in a high-risk population.
   - **Recommendation:** Refine the title to clearly emphasize the connection between ABO blood groups, COVID-19 severity, and the study’s biological focus (e.g., endothelial and adipocyte activation).
     - **Example:** "ABO Blood Group and COVID-19 Severity: Associations with Endothelial and Adipocyte Activation in Critically Ill Patients."
2. **Specify Study Type or Scope:**
   - **Current Title:** The title does not specify the study’s retrospective nature or population scope. Indicating the study type adds clarity, making it easier for readers to understand the research approach and scope.
   - **Recommendation:** Consider adding "Retrospective Study" or "Critically Ill Patients" to clarify the scope and nature of the research.
     - **Example:** "Retrospective Analysis of ABO Blood Group Associations with Endothelial and Adipocyte Activation in Critically Ill COVID-19 Patients."

**Review of the Abstract**

The abstract is crucial for summarizing the study’s background, methods, key findings, and conclusions in a way that is both comprehensive and accessible. Currently, it appears to provide an overview but could benefit from clearer focus, enhanced emphasis on key findings, and specific mention of study limitations and implications.

1. **Clarify Study Aim and Hypothesis:**
   - **Current:** The abstract briefly mentions ABO blood groups in relation to COVID-19 without clearly stating the study’s primary hypothesis. A focused introduction to the study aim helps readers understand the purpose and relevance of investigating ABO blood groups and these specific biological mechanisms.
   - **Recommendation:** Begin with a clear statement of the study’s aim, mentioning why ABO blood groups may influence COVID-19 severity and specific biological mechanisms being investigated (e.g., endothelial and adipocyte activation).
     - **Example:** "This study aimed to explore whether ABO blood groups are associated with COVID-19 severity in critically ill patients by examining their influence on endothelial and adipocyte activation markers."
2. **Describe the Population and Setting in Methods:**
   - **Current:** The methods section mentions the sample size but lacks details on the population and setting. Adding population and setting details enhances the specificity and relevance of the methods section, aiding in understanding the study context.
   - **Recommendation:** Specify the study’s population (e.g., critically ill COVID-19 patients) and setting (e.g., ICU at a specific hospital) to contextualize the findings.
   - **Example:** "A retrospective analysis was conducted on 128 critically ill COVID-19 patients admitted to [hospital] ICU, assessing associations between ABO blood groups, clinical outcomes, and biomarker levels."
3. **Emphasize Key Results with Clear Comparative Language:**
   - **Current:** The results are presented but could be clarified, especially by using comparative language to specify differences between groups. Comparative statements and precise statistics make the results section more impactful and easier to interpret.
   - **Recommendation:** Use clear comparative statements (e.g., "Patients with A/AB blood groups showed a 50% higher risk of ICU mortality compared to B/O groups") and highlight the significance of findings related to each clinical outcome or biomarker.
     - **Example:** "Patients with A/AB blood groups had significantly higher ICU mortality (29% vs. 11%, *p* = 0.018), more ventilation requirements (71% vs. 52%, *p* = 0.041), and elevated adipsin levels compared to B/O groups."
4. **Discuss Study Limitations and Generalizability:**
   - **Current:** The abstract does not mention study limitations, which could lead readers to overgeneralize the findings. Acknowledging limitations provides balance, setting appropriate expectations for interpreting the findings.
   - **Recommendation:** Briefly mention the primary limitations, such as the retrospective design, sample size, or potential confounding factors, and note that further studies are required.
     - **Example:** "The study’s retrospective design and limited sample size suggest that findings should be interpreted cautiously, with further research needed to confirm these associations."
5. **Highlight Clinical Implications and Future Directions in the Conclusion:**
   - **Current:** The conclusion notes the association found but does not elaborate on potential clinical applications or future research directions. A clear mention of clinical implications and future research directions makes the conclusion more actionable and relevant.
   - **Recommendation:** Briefly mention how these findings could inform clinical practice (e.g., risk stratification) and suggest specific areas for further research, such as exploring the underlying biological mechanisms.
     - **Example:** "These findings suggest that ABO blood group stratification may aid in COVID-19 risk assessment, particularly in critically ill patients, and warrant further research to elucidate underlying biological mechanisms."

**Review of Introduction**

The introduction provides a comprehensive background on the relationship between ABO blood groups and COVID-19 susceptibility and severity, referencing previous studies and hypotheses about potential mechanisms. It covers essential points, such as the broader implications of ABO blood groups in other infectious diseases and the theoretical mechanisms by which blood type might influence COVID-19 outcomes. However, there are areas where the introduction could be made more concise, focused, and engaging for the reader:

1. **Focus on the Research Gap:**
   - **Current:** The introduction discusses multiple studies about ABO blood group susceptibility to various diseases, including COVID-19. However, the specific research gap addressed by this study could be more prominently stated.
   - **Recommendation:** Early on, clarify the research gap: While ABO blood groups have been associated with COVID-19 severity, the precise mechanisms remain unclear. This study seeks to address this by analyzing specific biomarkers in critically ill patients. Emphasize this purpose to immediately set the study apart.
2. **Streamline Literature Background:**
   - **Current:** The introduction provides an extensive review of studies linking ABO blood types to COVID-19 and other infections, which may be overwhelming.
   - **Recommendation:** Focus on studies directly related to COVID-19 and blood groups, summarizing key findings in fewer sentences. Use transitional phrases to show how the literature review builds to the present study’s rationale.
3. **Organize Hypotheses and Mechanistic Theories:**
   - **Current:** The introduction discusses two primary hypotheses (the anti-A theory and ABO’s influence on systemic inflammation) but without clear emphasis or structure.
   - **Recommendation:** Introduce the hypotheses in separate, concise paragraphs, highlighting their relevance to COVID-19 severity. Then, relate these hypotheses directly to the markers studied (inflammatory cytokines, endothelial markers, and adipokines) to give the reader a clear sense of why these specific biomarkers were selected.
4. **Clarify Study Aims and Objectives:**
   - **Current:** The objectives are stated but could be more clearly connected to the research gap.
   - **Recommendation:** After describing the background and hypotheses, restate the study objectives explicitly. For example, “This study aims to (1) confirm the association between ABO blood groups and COVID-19 severity in critically ill patients and (2) explore potential mechanisms through biomarkers, focusing on inflammation, endothelial injury, and adipocyte activation.”
5. **Reduce Redundancy and Improve Flow:**
   - **Current:** Certain ideas are repeated, such as COVID-19 severity and ABO blood groups' association with other diseases.
   - **Recommendation:** Streamline repeated points by condensing related information into single, impactful sentences. This will make the introduction shorter and more direct, enhancing readability.
6. **Contextualize the Importance of Biomarkers:**
   - **Current:** The biomarkers are mentioned, but the introduction does not fully explain why they were chosen in the context of COVID-19 and ABO blood groups.
   - **Recommendation:** Briefly explain the relevance of each biomarker category (cytokines, endothelial markers, adipokines) to the study’s goal. For instance, highlight that these markers provide insights into inflammation and vascular responses, which are critical in COVID-19 pathology and potentially influenced by blood type.

**Review of the Methods Section**

The methods section effectively describes the study design, patient selection criteria, data collection methods, and statistical analyses used. However, several areas could benefit from more detail and clarification to enhance reproducibility and ensure that readers can fully understand and evaluate the study’s methodology.

1. **Patient Selection and Enrollment:**
   - **Current:** The section describes that 128 critically ill COVID-19 patients were enrolled but lacks specific inclusion and exclusion criteria details.
   - **Recommendation:** Provide explicit inclusion and exclusion criteria. For instance:
     - **Inclusion Criteria:** Patients admitted to the ICU for COVID-19 pneumonia, confirmed by a positive SARS-CoV-2 PCR test.
     - **Exclusion Criteria:** Patients with COVID-19 as an incidental finding or nosocomial infection, patients admitted >10 days post-ICU entry, and those already on immunomodulatory therapies upon ICU admission.
2. **Clinical and Biomarker Data Collection:**
   - **Current:** Data points (demographics, interventions, biomarkers) are mentioned but lack a clear description of the timing and handling of sample collection.
   - **Recommendation:** Detail the collection process, including:
     - **Sample Timing:** State that all biomarker samples were collected within a set timeframe (e.g., within 24 hours of ICU admission).
     - **Sample Processing and Storage:** Describe storage conditions (e.g., plasma samples stored at -80°C until analysis).
     - **Intervention Recording:** Specify how and when clinical interventions (e.g., ventilation) were recorded to ensure consistency across participants.
3. **Laboratory Assays and Biomarkers:**
   - **Current:** The biomarker assays are listed without sufficient details on assay validation and any potential modifications.
   - **Recommendation:** For each biomarker assay:
     - Specify assay names, vendors, and catalog numbers.
     - Include details on quality control measures, assay sensitivity, and intra-/inter-assay coefficients of variation.
     - If standard protocols were modified, clarify those changes to aid reproducibility.
4. **Definition of Clinical Outcomes:**
   - **Current:** The outcomes (mortality, ICU length of stay, need for ventilation) are defined but could benefit from more precise operational definitions.
   - **Recommendation:** Clearly define each outcome metric, for example:
     - **Mortality:** Define if this refers to all-cause ICU mortality within a specified timeframe.
     - **ICU Length of Stay:** Clarify whether this refers to total days spent in ICU or days from ICU admission to first discharge.
     - **Ventilation Requirements:** Specify if mechanical ventilation includes only intubated patients or also non-invasive ventilation.
5. **Control for Confounding Variables:**
   - **Current:** Although age and sex adjustments are mentioned in the statistical analysis, other potential confounders (e.g., comorbidities, time to ICU admission) are not discussed.
   - **Recommendation:** Consider adjusting for additional potential confounders that could influence COVID-19 severity, such as:
     - **Comorbidities:** Include variables like hypertension, diabetes, and BMI.
     - **ICU Admission Time:** Control for time from symptom onset to ICU admission.
6. **Statistical Analysis:**
   - **Current:** The methods mention Mann-Whitney U and Fisher’s exact tests without justifying the statistical approach.
   - **Recommendation:**
     - Explain the choice of statistical tests, especially why non-parametric tests were selected.
     - Provide more detail on logistic regression, such as if it’s binary or multinomial and which covariates were included.
     - Report any sensitivity analyses conducted to test the robustness of the findings.
7. **Ethical Considerations and Data Availability:**
   - **Current:** The study mentions ethical approval but lacks detail on patient data protection and data-sharing policies.
   - **Recommendation:**
     - Provide a statement on how patient data confidentiality was maintained and the use of anonymized data.
     - Clearly state any data-sharing plans, including public repository details if applicable.
8. **Justify the Stratification of Blood Groups into A/AB and B/O:**

- **Current:** The methods section describes the stratification of patients by blood group but does not provide a clear rationale for combining A with AB and B with O.
- **Recommendation:** Include a brief explanation of the biological and clinical rationale behind this grouping. For instance, the authors could explain that stratifying into A/AB and B/O was based on:
  - **Anti-A Antibody Hypothesis:** Blood groups B and O produce anti-A antibodies, which may offer a protective effect against SARS-CoV-2 infection by potentially hindering viral binding and entry. Grouping patients in this way allows for testing the hypothesis that anti-A antibodies influence COVID-19 severity.
  - **Coagulation and Thrombotic Risk Profiles:** Blood groups A and AB are linked with higher baseline levels of coagulation factors, which may increase thrombotic risk, a critical aspect of COVID-19 pathology. By grouping patients accordingly, the study aims to explore whether thrombotic and inflammatory differences associated with these blood groups contribute to variations in COVID-19 outcomes.

**Review of the Results Section**

The results section effectively presents key findings, particularly the associations between ABO blood group stratifications and clinical outcomes such as ICU stay, mortality, and biomarker levels. However, improvements in data presentation, organization, and interpretability are recommended to strengthen this section, ensuring that readers can fully grasp the significance and reliability of the findings:

1. **Organize Results by Primary and Secondary Outcomes:**
   - **Current:** The results section presents clinical and biomarker data but could benefit from a clearer, structured outline.
   - **Recommendation:** Separate findings by primary outcomes (e.g., ICU mortality, need for ventilation, length of ICU stay) and secondary outcomes (e.g., biomarker analyses).
     - **Primary Outcomes:** Include all data on patient outcomes, such as ventilation needs, ICU stay duration, and mortality rates.
     - **Secondary Outcomes:** Present biomarker findings (cytokines, endothelial markers, adipokines) under a separate heading.
2. **Clarify Stratification Results for Blood Groups A/AB and B/O:**
   - **Current:** The results mention differences between A/AB and B/O groups without discussing the biological rationale behind these differences.
   - **Recommendation:** Briefly summarize why differences between these groups are being highlighted, linking back to the study’s hypotheses.
     - For example: "Results showed that patients with A/AB blood groups had significantly higher ICU mortality and ventilation requirements compared to B/O, aligning with hypotheses regarding thrombotic risk and the potential protective role of anti-A antibodies in B/O patients."
3. **Include Detailed Summary Statistics in Tables:**
   - **Current:** The results section presents p-values and comparisons but lacks detailed summary statistics (e.g., medians, interquartile ranges, odds ratios). Adding these statistics in tables provides context for the magnitude of the effects and helps readers gauge clinical relevance beyond p-values alone.
   - **Recommendation:** For each major result, provide comprehensive summary statistics in tables, including:
     - Median, interquartile range, and odds ratios for categorical outcomes (e.g., ICU mortality, ventilation).
     - Confidence intervals for comparisons, particularly where statistical significance is noted.
4. **Enhance Biomarker Data Presentation:**
   - **Current:** Biomarker results are mentioned in the text without visual aids. Visual presentation of biomarker data will make differences or lack thereof more immediately apparent to readers and aid in comparing values across groups.
   - **Recommendation:** Present biomarker data with clearly labeled bar charts or box plots to illustrate differences between A/AB and B/O groups.
     - Use separate panels for each biomarker category (e.g., cytokines, endothelial markers, adipokines).
     - Highlight statistically significant findings with annotations on the graphs.
5. **Report Effect Sizes and Notable Non-Significant Findings:**
   - **Current:** The results section briefly mentions non-significant findings but does not elaborate. Reporting on trends in non-significant findings provides a more balanced view of the data and can guide future research directions
   - **Recommendation:** Include effect sizes for non-significant results and discuss trends where relevant. For instance:
     - Report if certain biomarkers approached significance or showed notable trends that could be explored in future research.
     - State whether the lack of association was consistent across all markers or varied by specific subtypes (e.g., no significant findings among all endothelial markers).
6. **Explain Handling of Missing or Excluded Data:**
   - **Current:** The results do not clarify whether any data points were excluded or how missing data were handled. Transparency about data handling practices enhances confidence in the results and ensures reproducibility.
   - **Recommendation:** Clearly indicate if any patients or data points were excluded from specific analyses due to missing data or other reasons.
     - State the number of participants analyzed for each biomarker, especially if it varied due to sample availability.
7. **Discuss Clinical Relevance of Findings in Brief Statements:**
   - **Current:** The section provides results without interpreting their clinical relevance. Briefly noting clinical relevance as each finding is presented can help readers immediately understand its potential impact on patient management.
   - **Recommendation:** Add brief statements to highlight the clinical implications of key findings. For example:
     - "The observed increase in ICU mortality among A/AB patients suggests a potential role for ABO blood group stratification in identifying high-risk patients."
8. **Limitations of Results Interpretation:**
   - **Current:** The results section does not mention any limitations of the data interpretation. Acknowledging limitations in the results section allows for a balanced interpretation and highlights areas where future research can expand on the findings.
   - **Recommendation:** Include a brief mention of limitations, such as the small sample size or potential effects of confounding variables.
     - For example: "While the findings suggest associations between ABO blood group and COVID-19 severity, the study’s observational nature limits causal inference, and additional studies are needed to confirm these results."

**Recommendations for Data Presentation and Clarity**

1. **Use Consistent Formatting Across Tables and Figures:**
   - **Current:** If there are inconsistencies in font size, alignment, or labeling across tables and figures, they can distract readers and make comparisons difficult. Consistent formatting improves the professional appearance of the results section and allows for smoother navigation and comparison across different data presentations.
   - **Recommendation:** Ensure that tables and figures have consistent formatting, including font style, size, and labeling. Align values (e.g., left-align text, center-align numbers) to make the data visually cohesive. Label each table and figure clearly with descriptive titles and concise legends.
2. **Provide Additional Context with Each Figure and Table Legend:**
   - **Current:** Legends may be brief, limiting readers' ability to interpret the data without reading the entire results section. Detailed legends allow readers to understand the key takeaway from each visual presentation at a glance, without needing to refer to the main text.
   - **Recommendation:** Add brief descriptions in each legend to explain what the figure or table represents and what comparisons are being highlighted.
     - For example: “Figure 1: Comparison of ICU mortality rates between A/AB and B/O blood groups in critically ill COVID-19 patients, showing a significant increase in mortality for A/AB patients.”
3. **Highlight Key Findings in Tables with Bold Text or Shading:**
   - **Current:** Important values may not stand out, making it difficult for readers to identify key findings quickly. Visual emphasis on critical values draws readers’ attention to the most important findings, improving data accessibility and interpretability.
   - **Recommendation:** Use bold text, italics, or subtle shading to highlight statistically significant or clinically relevant values in tables.
4. **Add Subgroup Analyses Tables for Biomarkers if Relevant:**
   - **Current:** Biomarker data may be presented without considering subgroup differences (e.g., age, comorbidities). Subgroup analyses can reveal nuanced differences within the dataset, allowing a more granular understanding of biomarker associations in different patient populations.
   - **Recommendation:** If feasible, include tables or figures that show biomarker levels in relevant subgroups, such as age groups or presence of specific comorbidities. This could include separate columns or rows in tables.
5. **Add Summary Visuals to Highlight Key Biomarker Differences:**
   - **Current:** If the biomarker data are complex and scattered, it may be hard for readers to capture overall trends. A summary visualization for biomarkers provides an at-a-glance view of all analyzed markers, making it easier to detect patterns across blood groups.
   - **Recommendation:** Consider adding a heatmap or clustered bar chart showing relative levels of each biomarker across groups, with color intensity indicating higher or lower levels.
6. **Provide Interpretation Notes for Non-Significant Findings:**
   - **Current:** Non-significant findings are often presented without context, potentially leading to misinterpretation. Annotating non-significant findings helps readers understand that these markers were examined and not associated with blood group differences, guiding future research directions.
   - **Recommendation:** Briefly annotate figures or tables with non-significant findings, providing insight such as “No significant difference observed in IL-6 levels between A/AB and B/O groups.”
7. **Ensure Tables and Figures are Self-Explanatory:**
   - **Current:** Readers may need to refer to the text to understand what each table or figure represents. Making each table and figure self-contained allows readers to understand the content independently, enhancing the section’s overall clarity.
   - **Recommendation:** Add sufficient explanatory details within each table and figure (e.g., footnotes explaining abbreviations, units of measurement, or grouping variables).
8. **Use a Uniform Scale for Comparable Figures:**
   - **Current:** Figures may have different y-axis scales, making it hard to compare data across figures. Standardizing the y-axis across comparable figures makes it easier for readers to visually compare results, reducing confusion.
   - **Recommendation:** For all figures that compare similar metrics (e.g., patient outcomes, biomarker levels), use a consistent y-axis scale. If certain variables require a different scale due to extreme values, add a note explaining why.
9. **Add Clear Legends and Labels with Units:**
   - **Current:** If units or scales aren’t consistent across figures, readers may struggle to compare values accurately. Consistent labeling with units clarifies what each bar represents, allowing for more accurate and immediate comparisons.
   - **Recommendation:** Clearly label each figure with units and ensure legends specify the data type (e.g., percentage, count, median levels).
10. **Implement Reference Lines for Key Metrics:**
    - **Current:** Without reference points, bar heights can be hard to contextualize. Reference lines provide a visual anchor, allowing readers to see at a glance which groups fall above or below the average.
    - **Recommendation:** For figures showing key outcomes or biomarkers, consider adding reference lines (e.g., average or median line across all groups) to make comparisons easier.

**Review of the Discussion Section**

The discussion section successfully covers the main findings and their implications but could benefit from more detailed analysis, particularly in addressing study limitations and integrating relevant literature. Enhancing the discussion with a structured approach, addressing potential confounding factors, and providing a clearer interpretation of results in the context of sample size limitations and stratification choices will strengthen the section.

1. **Provide a Clear Summary of Key Findings and Hypotheses:**
   - **Current:** The discussion begins with a description of the primary results but does not adequately connect them back to the study’s hypotheses. A clear opening summary helps set the tone for the discussion and contextualizes each finding in relation to the study’s goals.
   - **Recommendation:** Start with a concise summary of key findings, directly linking them to the hypotheses about anti-A antibodies, thrombosis, and inflammatory markers.
     - For example: “This study found that patients with A/AB blood groups had significantly higher ICU mortality and ventilation requirements than B/O groups, consistent with our hypothesis that anti-A antibodies may play a protective role.”
2. **Interpret the Findings in Light of Previous Literature:**
   - **Current:** The discussion briefly mentions related studies but does not fully integrate them to build a case around the observed findings. Integrating these studies provides a robust scientific basis for the findings and highlights where the study’s results align or differ from existing research.
   - **Recommendation:** Cite recent studies that provide context for the protective role of anti-A antibodies and ABO blood group influences on thrombotic and inflammatory responses in COVID-19.
     - For example:
       - **Anti-A Antibodies:** Refer to studies that suggest anti-A antibodies can interfere with SARS-CoV-2 binding, possibly explaining the reduced severity in B/O groups (e.g., Guillon et al., 2008; Zhao et al., 2020).
       - **Thrombotic Risk and ABO Groups:** Cite literature supporting blood group-related differences in coagulation factors, which may account for increased thrombotic complications in A/AB patients (e.g., Ellinghaus et al., 2020; Conway et al., 2022).
3. **Discuss Potential Confounders in Depth:**
   - **Current:** The discussion lacks an in-depth analysis of potential confounders (e.g., comorbidities, treatment variations). Addressing confounders shows a comprehensive approach to data interpretation and strengthens the reliability of the observed associations
   - **Recommendation:** Address potential confounders that might influence COVID-19 severity and ABO associations, such as:
     - **Comorbidities** (e.g., hypertension, diabetes, obesity) that are more prevalent in certain blood groups.
     - **Variations in Treatment Protocols**, such as the timing of corticosteroid or antiviral therapy, which could impact mortality and ICU outcomes.
   - Consider if these confounders were adjusted for and clarify any limitations in controlling them.
4. **Explain and Justify the A/AB and B/O Group Stratification in the Discussion:**
   - **Current:** The stratification is mentioned in results but not thoroughly justified in the discussion. Reinforcing the stratification rationale helps clarify the choice and shows a thoughtful approach to analyzing blood group effects on COVID-19 severity.
   - **Recommendation:** Reiterate the rationale for combining A/AB and B/O groups and discuss how this grouping aligns with the study’s hypotheses.
     - Highlight that grouping A with AB (lacking anti-A antibodies) and B with O (having anti-A antibodies) was intentional to explore the protective effects of anti-A antibodies.
     - Address any limitations this stratification might impose on detecting blood group-specific effects (e.g., unique effects in group AB).
5. **Address Sample Size Limitations and Statistical Power:**
   - **Current:** The discussion does not fully address the potential impact of sample size on findings, particularly in biomarker analysis. Addressing sample size limitations conveys transparency and underscores the exploratory nature of the findings, making room for future validation studies.
   - **Recommendation:** Acknowledge that the sample size may limit the statistical power to detect smaller but potentially clinically relevant associations, especially in biomarker analyses.
     - Discuss how limited sample sizes could affect the generalizability of findings and suggest that future studies with larger cohorts are needed to confirm these results.
6. **Explore Alternative Explanations and the Null Findings for Biomarkers:**
   - **Current:** Some biomarkers showed no significant differences across groups, but these results are not discussed in depth. Exploring null findings adds balance to the discussion, demonstrating that the authors considered and investigated potential reasons for these non-significant results.
   - **Recommendation:** Discuss why certain biomarkers (e.g., IL-6, endothelial markers) did not vary by ABO group, offering alternative explanations.
     - Mention that these biomarkers may not directly mediate the observed differences in clinical outcomes, or that their dynamic nature in COVID-19 could obscure group differences.
7. **Link Biomarker Findings to Clinical Relevance and Future Research:**
   - **Current:** The discussion briefly mentions adipsin as a potentially significant biomarker but lacks depth on clinical implications. Highlighting the clinical relevance of adipsin and other biomarkers helps underscore the study’s contributions and directs future research.
   - **Recommendation:** Expand on the potential role of adipsin in COVID-19 pathology and its relevance to blood group differences.
     - Suggest that adipsin could be a biomarker for more severe disease in A/AB groups and recommend further research to investigate its mechanistic role.
8. **Provide Practical Implications and Potential for Clinical Application:**
   - **Current:** The practical implications of the findings for clinical practice are not well articulated. Offering practical implications connects the research findings to real-world applications, enhancing the study’s relevance for healthcare providers.
   - **Recommendation:** Discuss how the observed associations between ABO blood groups and COVID-19 severity might be useful in clinical settings.
     - For example, suggest that blood group stratification might aid in risk assessment or resource allocation in ICU settings.
9. **Acknowledge Broader Limitations and Scope for Future Research:**
   - **Current:** Limitations are only briefly mentioned. A comprehensive limitations section shows a balanced perspective on the findings and acknowledges where additional research is necessary to build on these results.
   - **Recommendation:** Conclude with a section on broader limitations and recommendations for future research:
     - **Study Design and Retrospective Nature:** Note that the retrospective design limits causal inference.
     - **Diverse Populations:** Recommend replicating the study in diverse populations, as genetic and environmental factors could influence the generalizability of ABO effects on COVID-19.
     - **Extended Biomarker Analysis:** Suggest future studies with a broader biomarker panel and more precise timing of sample collection to capture dynamic changes.

**Recommendations for Discussing Results in Comparison to International Studies**

1. **Highlight Consistencies and Variations Across Studies:**
   - **Current:** The discussion lacks a comparison of findings from other countries, which limits the ability to understand whether the results are consistent globally or specific to the study's population. Comparing findings from various countries enhances understanding of whether the observed associations between ABO blood groups and COVID-19 outcomes are universally applicable or region-specific, influenced by factors like genetic diversity or healthcare systems.
   - **Recommendation:** Integrate comparisons to studies from different countries to highlight whether the findings align with global patterns or show unique trends. Specifically, discuss how results from international studies on ABO blood group distribution and COVID-19 outcomes compare to the findings in this study.
2. **Compare Findings to Studies Listed by the Reviewer:**
   - **Study 1:** **Reference:** [*https://doi.org/10.2174/1389203723666220811121803*](https://doi.org/10.2174/1389203723666220811121803)
     - **Context:** This study explores the relationship between ABO blood groups and COVID-19 severity in a different population.
     - **Recommendation:** Discuss whether the association between A/AB blood groups and increased COVID-19 severity aligns with findings from this study. Note any differences in demographic or healthcare factors that might explain discrepancies.
   - **Study 2:** **Reference:** [*https://doi.org/10.3389/bjbs.2022.10098*](https://doi.org/10.3389/bjbs.2022.10098)
     - **Context:** This study examines ABO blood groups in relation to COVID-19 in another geographic region.
     - **Recommendation:** Highlight any commonalities in the protective role of anti-A antibodies (as seen in B/O groups) across regions, or address discrepancies in outcomes. Discuss potential reasons for these differences, such as genetic background or environmental influences.
   - **Study 3:** **Reference:** *10.1097/MD.0000000000028334*
     - **Context:** This study addresses how ABO blood group variations affect COVID-19 outcomes.
     - **Recommendation:** Compare specific outcomes, such as ICU stay duration, ventilation needs, and mortality rates, to determine if these markers show similar patterns in different populations. If not, suggest factors that might explain these differences.
   - **Study 4:** **Reference:** [*https://doi.org/10.1016/j.transci.2021.103169*](https://doi.org/10.1016/j.transci.2021.103169)
     - **Context:** This study examines ABO blood group impacts on susceptibility COVID-19.
     - **Recommendation:** Discuss how findings on blood group-specific risk factors might explain differences in clinical outcomes. Address any transfusion-related recommendations and whether they align with or differ from international recommendations.
   - Including these specific studies provides a comprehensive global perspective, showing where findings are consistent or divergent. This analysis allows for a deeper understanding of how blood group associations with COVID-19 severity may vary by location.
3. **Consider Demographic and Population Health Differences:**
   - **Current:** The discussion does not explore how population demographics may influence ABO-COVID-19 associations. Recognizing demographic and health system differences provides a nuanced view, emphasizing that findings may vary based on the population studied.
   - **Recommendation:** Address how factors like population genetics, health system differences, and demographic variations (e.g., age distribution, prevalence of comorbidities) might contribute to differences in ABO associations observed across countries.
4. **Discuss Potential Genetic and Environmental Influences:**
   - **Current:** The authors do not address possible genetic or environmental factors affecting results. Exploring genetic and environmental influences provides a more comprehensive understanding of factors that may modify blood group effects on COVID-19 severity.
   - **Recommendation:** Mention that genetic variation in ABO blood group distribution and environmental factors (e.g., diet, climate) might contribute to the differences observed in COVID-19 outcomes across countries.
5. **Acknowledge Limitations in Cross-Country Comparisons:**
   - **Current:** There is no mention of limitations in making cross-country comparisons. Recognizing these limitations makes the discussion more balanced and ensures that readers interpret cross-country comparisons with appropriate caution.
   - **Recommendation:** Acknowledge that differences in study design, population structure, healthcare practices, and data collection methods can impact cross-country comparisons.

**Review of the Conclusion Section**

The conclusion section summarizes the study’s key findings regarding the association between ABO blood groups and COVID-19 severity. While it provides an overview of the observed associations and suggests areas for future research, it could be improved by offering a more focused summary, discussing clinical implications, and addressing study limitations.

1. **Emphasize Key Findings with a Clear and Concise Summary:**
   - **Current:** The conclusion summarizes the results but could be more focused on the main takeaways. A concise summary makes the conclusion more impactful and allows readers to immediately grasp the study’s most important findings.
   - **Recommendation:** Condense the key findings into 1-2 sentences that specifically highlight the association between A/AB blood groups and more severe COVID-19 outcomes (e.g., higher ICU mortality, ventilation needs).
     - Example: "This study found that COVID-19 patients with A/AB blood groups experienced higher ICU mortality and greater ventilation requirements than those with B/O groups, potentially due to the protective effects of anti-A antibodies and differences in thrombotic risk."
2. **Discuss Clinical Implications and Potential Applications:**
   - **Current:** The conclusion mentions the need for further research but does not explore the practical implications of these findings. Adding clinical implications enhances the relevance of the research findings for healthcare providers and may encourage practical applications.
   - **Recommendation:** Briefly discuss how these findings could inform clinical practice, such as by suggesting that ABO blood groups might help in identifying high-risk patients or guiding ICU resource allocation.
     - Example: "If confirmed in larger studies, ABO blood group stratification could be a valuable tool for early risk assessment in critically ill COVID-19 patients, aiding in resource prioritization and personalized care."
3. **Acknowledge Study Limitations:**
   - **Current:** The conclusion does not address limitations, which may leave readers with an overly optimistic interpretation of the findings. Addressing limitations demonstrates transparency and strengthens the credibility of the findings, providing a balanced perspective.
   - **Recommendation:** Include a brief acknowledgment of key limitations, such as the observational design, limited sample size, and potential for confounding factors.
     - Example: "The observational nature and limited sample size of this study restrict causal inference, and future research should adjust for additional confounders to confirm these associations."
4. **Propose Specific Directions for Future Research:**
   - **Current:** The conclusion mentions the need for more research but lacks specificity. Specific recommendations provide a clear roadmap for further investigation, emphasizing areas where research could deepen our understanding of ABO blood group impacts on COVID-19.
   - **Recommendation:** Offer targeted suggestions for future studies, such as investigating specific biomarkers, larger-scale studies across diverse populations, or exploring mechanistic links between ABO blood groups and inflammatory pathways in COVID-19.
     - Example: "Future studies should examine these associations in larger, multi-center cohorts and further investigate the role of specific inflammatory and thrombotic biomarkers in mediating ABO-dependent COVID-19 severity."
5. **Avoid Overstating the Findings:**
   - **Current:** The conclusion may give the impression that the findings are definitive, potentially overstating the study’s impact. Cautious wording prevents overstating the study's impact and highlights the need for further validation, which aligns with the principles of scientific integrity.
   - **Recommendation:** Use cautious language to convey that the findings are preliminary and require validation.
     - Example: "While our results suggest an association between ABO blood groups and COVID-19 severity, these findings should be interpreted with caution and validated in larger, prospective studies."

**Review of the References Section**

The references section is an essential component, as it reflects the study's foundation and situates it within the broader scientific literature. The current references list provides relevant background but could be enhanced by including more recent and specific studies on COVID-19, ABO blood groups, and related biomarkers.

1. **Update with the Most Recent and Relevant Literature:**
   - **Current:** Some references may be general and lack the latest research on COVID-19 and ABO blood group associations. Citing the most current and relevant literature ensures that the study is anchored within the latest research, strengthening the rigor of the references section.
   - **Recommendation:** Include recent studies that are highly relevant to the study’s findings, especially those published in 2022 or later.
     - Examples to consider:
       - Studies on the protective role of anti-A antibodies in SARS-CoV-2 infection.
       - Research on ABO-related thrombotic and inflammatory responses in COVID-19 (e.g., Conway et al., 2022).
2. **Ensure a Balanced Representation of Supporting and Contrasting Studies:**
   - **Current:** The references may not fully capture contrasting findings or studies with different perspectives. Including contrasting studies demonstrates a comprehensive review of the literature, lending credibility to the discussion by acknowledging a range of perspectives.
   - **Recommendation:** Add references to studies with findings that do not support or are neutral about the ABO-COVID-19 association, providing a balanced scientific perspective.
3. **Include Specific References for Biomarker and Mechanistic Studies:**
   - **Current:** The references section lacks detailed citations for specific biomarkers discussed in the study (e.g., adipsin, IL-6). Citing biomarker-specific literature supports the study’s biomarker analysis and provides context for readers interested in the mechanistic basis of the findings
   - **Recommendation:** Reference specific studies that have explored the role of each biomarker in COVID-19 severity and its potential relationship with ABO blood groups.
4. **Ensure Consistent Formatting and Accuracy in Citations:**
   - **Current:** There may be inconsistencies in citation formatting or outdated references. Accurate and consistent formatting reflects attention to detail, enhancing the professionalism of the manuscript.
   - **Recommendation:** Check for any inconsistencies in formatting (e.g., author initials, journal abbreviations) and verify that all citations are current.
5. **Add Key Background Studies on Thrombotic and Inflammatory Pathways in COVID-19:**
   - **Current:** Some of the underlying mechanisms discussed, such as thrombotic and inflammatory pathways, may not be adequately supported by citations. These studies provide essential background, supporting mechanistic discussions and aligning with the study’s exploration of ABO-related thrombotic and inflammatory differences.
   - **Recommendation:** Cite foundational studies on COVID-19’s thrombotic complications, coagulopathy, and inflammation.
     - Example references:
       - Mehta et al., 2020, discussing the cytokine storm and its impact on COVID-19 outcomes.
       - Conway et al., 2022, on coagulopathy and the impact of thrombotic risk factors in COVID-19.
