# Supplementary material for: ABO blood group and COVID-19 severity: Associations with endothelial and adipocyte activation in critically ill patients
Source: PLoS One. 2025 Apr 2;20(4):e0320251. doi: 10.1371/journal.pone.0320251 (PMC11964209; doi:10.1371/journal.pone.0320251)
Supplement: Attachment — Submitted filename: Response to reviewers 2025 02 07.docx [file pone.0320251.s006.docx]

Response to reviewers

PONE-D-24-45720

Association of ABO Blood Group with Endothelial and Adipocyte Activation in COVID-19

Dear Dr. Patnaik

Thank you for sending our manuscript to 5 expert reviewers. We have carefully addressed all of their specific comments in the Table below. We hope this will meet with your satisfaction.

Warm regards

Sophie Stukas and Luke Y.C. Chen

PLOS ONE

Dear Dr. Y.C. Chen,

Thank you for submitting your manuscript to PLOS ONE. After careful consideration, we feel that it has merit but does not fully meet PLOS ONE’s publication criteria as it currently stands. Therefore, we invite you to submit a revised version of the manuscript that addresses the points raised during the review process.

Please submit your revised manuscript by Jan 27 2025 11:59PM. If you will need more time than this to complete your revisions, please reply to this message or contact the journal office at [plosone@plos.org](mailto:plosone@plos.org). When you're ready to submit your revision, log on to <https://www.editorialmanager.com/pone/> and select the 'Submissions Needing Revision' folder to locate your manuscript file.

Please include the following items when submitting your revised manuscript:

- A rebuttal letter that responds to each point raised by the academic editor and reviewer(s). You should upload this letter as a separate file labeled 'Response to Reviewers'.
- A marked-up copy of your manuscript that highlights changes made to the original version. You should upload this as a separate file labeled 'Revised Manuscript with Track Changes'.
- An unmarked version of your revised paper without tracked changes. You should upload this as a separate file labeled 'Manuscript'.

If you would like to make changes to your financial disclosure, please include your updated statement in your cover letter. Guidelines for resubmitting your figure files are available below the reviewer comments at the end of this letter.

If applicable, we recommend that you deposit your laboratory protocols in protocols.io to enhance the reproducibility of your results. Protocols.io assigns your protocol its own identifier (DOI) so that it can be cited independently in the future. For instructions see: [https://journals.plos.org/plosone/s/submission-guidelines#loc-laboratory-protocols](https://can01.safelinks.protection.outlook.com/?url=https%3A%2F%2Ftrack.editorialmanager.com%2FCL0%2Fhttps%3A%252F%252Fjournals.plos.org%252Fplosone%252Fs%252Fsubmission-guidelines%2523loc-laboratory-protocols%2F1%2F010f0193bf5691e5-0e04bde6-2e5e-4460-b29b-16ab3b2dd4c8-000000%2Fn793x9Oci7AbbxWUDyCxrqBPmVttspUKzIBsJY_btRI%3D189&data=05%7C02%7Clchen2%40bccancer.bc.ca%7C79ab83d7a1594d2fb2a308dd1b582a61%7C31f660a5192a4db392baca424f1b259e%7C0%7C0%7C638696788108591363%7CUnknown%7CTWFpbGZsb3d8eyJFbXB0eU1hcGkiOnRydWUsIlYiOiIwLjAuMDAwMCIsIlAiOiJXaW4zMiIsIkFOIjoiTWFpbCIsIldUIjoyfQ%3D%3D%7C0%7C%7C%7C&sdata=7bFnN%2BIRvAFnj94zgPWLI9AB0eAdv4W8m9e%2FAMBNjcI%3D&reserved=0). Additionally, PLOS ONE offers an option for publishing peer-reviewed Lab Protocol articles, which describe protocols hosted on protocols.io. Read more information on sharing protocols at [https://plos.org/protocols?utm_medium=editorial-email&utm_source=authorletters&utm_campaign=protocols](https://can01.safelinks.protection.outlook.com/?url=https%3A%2F%2Ftrack.editorialmanager.com%2FCL0%2Fhttps%3A%252F%252Fplos.org%252Fprotocols%253Futm_medium%3Deditorial-email%2526utm_source%3Dauthorletters%2526utm_campaign%3Dprotocols%2F1%2F010f0193bf5691e5-0e04bde6-2e5e-4460-b29b-16ab3b2dd4c8-000000%2FFtkdKIMbdou7O9OHMKuska5GsNPyFON1TTaIZIShy-o%3D189&data=05%7C02%7Clchen2%40bccancer.bc.ca%7C79ab83d7a1594d2fb2a308dd1b582a61%7C31f660a5192a4db392baca424f1b259e%7C0%7C0%7C638696788108614555%7CUnknown%7CTWFpbGZsb3d8eyJFbXB0eU1hcGkiOnRydWUsIlYiOiIwLjAuMDAwMCIsIlAiOiJXaW4zMiIsIkFOIjoiTWFpbCIsIldUIjoyfQ%3D%3D%7C0%7C%7C%7C&sdata=Q%2FeTOxl3Qft2W4844a%2Ba2iCveslrI68%2B62CPl%2B2bAXo%3D&reserved=0).

We look forward to receiving your revised manuscript.

Kind regards,

Santosh K. Patnaik, MD, PhD
Academic Editor
PLOS ONE

Additional Editor Comments:

The manuscript has been examined by five referees. I request that you kindly address all of their concerns through changes in the manuscript and/or comments in the Response to Review letter.


Reviewers' comments:

Reviewer's Responses to Questions

**Comments to the Author**

| Comment | Response |
| --- | --- |
| Reviewer 1 [Dr. Saeed M Kabrah] |  |
| This manuscript explores the association between ABO blood groups and COVID-19 severity, focusing on clinical outcomes in critically ill patients and the role of endothelial and adipocyte activation markers. The study addresses an important question about potential risk factors for severe COVID-19 outcomes, and the findings on blood group A/AB being associated with higher ICU mortality and ventilation requirements are intriguing and could have implications for clinical risk stratification. However, certain areas require clarification and enhancement to improve the manuscript’s clarity, scientific rigor, and relevance to a global audience. | Thank you please see specific responses below |
| Title Suggestions: The current title, “Association of ABO Blood Group with Endothelial and Adipocyte Activation in COVID-19,” could be made more descriptive. Consider revising the title to capture both the critical patient population (critically ill COVID-19 patients) and the study’s specific objectives (COVID-19 severity, endothelial/adipocyte activation). A suggested title might be, “ABO Blood Group and COVID-19 Severity: Associations with Endothelial and Adipocyte Activation in Critically Ill Patients.” | Thank you, we have changed the title to: “ABO Blood Group and COVID-19 Severity: Associations with Endothelial and Adipocyte Activation in Critically Ill Patients” as suggested |
| **abstract** |  |
| Abstract Improvements: Study Aim and Hypothesis: Begin with a clear statement of the study aim and the hypothesis that ABO blood group associations with COVID-19 severity might be mediated through specific biological mechanisms. | Thank you we changed the 2^nd^ sentence in the Background to: The aim of this study was to explore a potential association between ABO blood group and severity of COVID-19 infection in critically ill patients and the following biological mechanisms: inflammatory cytokines, endothelial injury, and adipokines. |
| Results Presentation: Use comparative language to clarify differences between groups. For instance, specify that patients with A/AB blood groups showed higher ICU mortality (29% vs. 11%) and more frequent ventilation needs (71% vs. 52%).  Limitations and Future Research: Mention the retrospective nature and limited sample size as key limitations in the abstract, along with a note on the need for further studies to confirm these associations and investigate underlying mechanisms. | Thank you, we changed the first sentence to read: “The association between ABO and severity of disease was confirmed. Patients with A&AB blood group had **more frequent** ventilation requirements compared to patients with blood group B&O ( N(%): 35 (71%) vs 41 (52%), p=0.041),”  We changed the Conclusions: section to reflect these comments:  This **retrospective single-center study** confirms an association between A and AB blood type with more severe COVID-19. While an underlying mechanism was not identified, the finding of higher adipsin levels in patients with type A/AB blood warrants further investigation **in larger prospective studies.** |
| Introduction and Background The introduction provides useful context on ABO blood groups and general COVID-19 pathology but would benefit from a clearer statement of the study’s specific hypothesis and objectives related to endothelial and adipocyte activation. Consider adding recent references to further strengthen the literature review, particularly regarding the protective effects of anti-A antibodies in B/O groups and the potential role of thrombotic risk factors associated with blood groups. | Thank you – please see the response to the comment 7 rows down (“Discuss the results in the context of international studies…”). We have added the references you suggested (thank you for these) |
| Methods Section Clarify Patient Selection and Data Collection:  Explicitly state inclusion and exclusion criteria and provide more details on data collection timing and handling to enhance reproducibility. | Thank you, the inclusion and exclusion criteria are stated explicitly on page 6, as follows:  **Study Design and Participants:** Patients in the present study were enrolled as part of a prospective COVID-19 biomarker study.[41,42] Adult patients admitted to the intensive care unit (ICU) at the Vancouver General Hospital with a diagnosis of pneumonia secondary to SARS-CoV-2 infection between March 30, 2020 and March 31, 2021 were included. Patients were excluded if: COVID-19 was an incidental finding on or during admission (e.g. admitted to ICU primarily for trauma), if COVID-19 was determined to be nosocomial in origin, or if study enrollment took place >10 days after initial ICU admission. |
| Consider including information on quality control for biomarker assays and any adjustments made for potential confounders (e.g., comorbidities, treatment variations). | Thank you for your suggestion. We have added the following information to the methods section detailing the biomarker analysis to include the experimental design and within sample variability as follows [ page 8 ] :  “*Each assay plate contained an 8-point calibrator curve and 2-3 control samples to monitor inter-plate variability (inflammatory analysis only). All samples were analyzed in duplicate, with the average of the values reported, in a randomized order. The average intra-sample CV was: Human Coagulation Panel 3 4-Plex Assay 8-20%; Human Cardiovascular Disease Panel 2 2-Plex 14-19%; Human Cardiovascular Disease Panel 4 1-Plex 9%; Human Adipokine Panel 1 5-Plex Assay 9-26%; Simoa Cytokine 3-Plex Assay 5-6%; Simoa IL-1β assay 14%; and sIL-6R and sgp130 8%.”*  The groups were balanced for known confounders such as sex,age, bmi, as shown in table 1 |
| Rationale for Group Stratification: Justify the stratification into A/AB vs. B/O groups by explaining the relevance of anti-A antibodies and thrombotic risk differences. Consider the limitations of combining blood groups in this way and address how it may affect the interpretation of findings. | Thank you, we compared A/AB vs B/O for the following reasons:   1) Consistency with our prior publication, for which the present study provides a larger cohort for confirmation (Hoiland Blood Advances 2020, reference 6) 2) We wanted to test the “anti-A” hypothesis and our sample size was not sufficient to divide separately into A, AB, B, and O for any meaningful comparison. |
| Statistical Analysis: Provide more details on statistical methods used, including reasons for selecting non-parametric tests and any adjustments made for confounding factors. Consider including sensitivity analyses to confirm the robustness of findings. | We are happy to provide clarification on the statistical methods used. Non-parametric tests were used as the vast majority (and in essence all of the biomarker data) is not normally distributed. For groupwise biomarker comparison, we did not make any adjustments for potential confounding factors for the reasons described in our response above and the fact that this was a secondary analysis of a cohort, thus we were limited in the study design elements that could be taken into account post-hoc. The statistical section of the methods now reads [ page 8 ] :  “*Data normality was assessed using a Sharpiro-Wilk test and Q-Q plots. As biomarker data was almost exclusively non-normally distributed, continuous data were described using median and interquartile range (IQR) and group differences between participants with blood group A or AB were compared to participants with blood group B or O using a non-parametric Mann-Whitney U test. Categorical variables were described using N and frequency and group differences were tested using a Fisher’s exact test. For categorical clinical outcomes, such as the need for mechanical ventilation, CRRT, or ECMO and mortality, odds ratios (OR) were determined using a logistic regression model corrected for age and sex*.”  Regarding sensitivity analysis – as our results are more exploratory in nature and we are limited in term of cohort size, we did not do any further sensitivity analysis. We have included text within the discussion to highlight that future studies are need to confirm whether our observations are generalizable. |
| Results Section Standardize Data Presentation:  Use consistent y-axis scales and bar lengths across figures to improve readability. Present summary statistics (e.g., medians, interquartile ranges, odds ratios) in tables for clarity. | Thank you for your comment. The Y-axis are not all consistent as there is a considerable variation in the concentration across the broad array of analytes tested, and thus setting one range would make it very difficult to visualize the data. The bar lengths are defined as the interquartile range with whiskers set to min and max. For a description of the median, IQR, and p-value please see Supplemental Table 2 where this is listed. |
| Results: Emphasize Key Findings with Clear Comparative Language: Use clear language to highlight significant results, such as ICU mortality and ventilation differences, and visually distinguish statistically significant values in tables and figures. Provide visual aids, like bar charts or box plots, to represent biomarker comparisons across groups, as these will improve readers’ ability to interpret differences. | To clarify our results we have added that the statistically significant differences in ventilation and mortality are a comparison directly between participants with blood group A or AB versus those with blood group O or B.  We have bolded significant results in tables to visually distinguish statistically values. Box and whisker plots are displayed in Figure 1-3 to display the compared values of key inflammatory, endothelial, and adipokine biomarkers compared between participants with A/AB vs O/B ABO group. |
| Discuss Non-Significant Findings and Trends: Briefly mention trends or non-significant findings, particularly in biomarker analyses, to give a balanced view of the results and suggest areas for future investigation. | Thank you, for the sake of brevity we have focused on significant findings. The non-significant findings are laid out clearly in the displays and the readers can draw their own interpretation for future investigation. |
| Discuss the results in the context of international studies, such as those in other populations or regions, to assess whether these findings may be generalizable. Specific studies include: <https://doi.org/10.2174/1389203723666220811121803> <https://doi.org/10.3389/bjbs.2022.10098> 10.1097/MD.0000000000028334  <https://doi.org/10.1016/j.transci.2021.103169>  Highlight differences that may arise from genetic, demographic, or environmental factors. Interpret Key Findings in Light of Hypotheses: | Thank you, we have referenced these studies  <https://doi.org/10.3389/bjbs.2022.10098> [ inserted – Intro para 4]  10.1097/MD.0000000000028334 [inserted Intro para 1]  <https://doi.org/10.1016/j.transci.2021.103169> [ inserted Intro para 1]  These differences related to genetic, demographic, environmental factors are mentioned in “Limitations section”, page 12: “Single center studies of COVID-19 are inherentily limited, as there are numerous genetic, environmental and demographic factors which likely affect outcomes in COVID-19” |
| Relate findings directly to the study’s hypotheses about anti-A antibodies and thrombotic risks associated with blood groups. Discuss any unexpected or null findings in biomarkers to provide a comprehensive view. | Thank you, references 24 and 25 in the Intro, page 4 address this point.  We discuss the negative and positive findings of the study throughout the Discussion section. |
| Acknowledge Limitations and Implications for Future Research:  Discuss limitations, such as the sample size, observational nature, and potential confounding factors. Suggest that future studies with larger, diverse populations and expanded biomarker analyses could further explore these associations. | Thank you, please see response two rows above. |
| Highlight Clinical Implications:  Briefly discuss how these findings could inform COVID-19 risk assessment or ICU resource allocation if validated in larger cohorts. Ethical Considerations  Dual Publication: No concerns about dual publication were noted, provided that the study’s content is original and not previously published in a similar format elsewhere. Data Confidentiality: Ensure that patient confidentiality is maintained, with no identifying information in figures or descriptions. Research Ethics Approval: Confirm that ethical approval was obtained for this study and explicitly mention it in the methods section. If applicable, discuss patient consent, particularly if data were collected retrospectively. | Thank you this is an original study that is part of a prospective biomarker protocol at UBC. The others studies published pertaining to this protocol are referenced in the Study Design and Participants section, p. 6  Thank you, this information is on page 6, Methods:  The study was approved by the University of British Columbia Clinical Research Ethics Board (UBC CREB, H20-00971) and registered on ClinicalTrials.gov (NCT04363008). Due to the retrospective nature of the present study, (University of British Columbia Clinical Research Ethics Board) waived the need for obtaining individual informed consent. The study data were accessed Mar 30, 2020 to Sep 30, 2024. All research was conducted in accordance with the principles of the Helsinki declaration and Strengthening the Reporting of Observational Studies in Epidemiology guidelines. |
| Provide a focused summary of the main findings, with a cautious interpretation due to the study’s limitations. Avoid overstating results and emphasize that further research is required to confirm these associations.  Suggest Specific Directions for Future Research: Include specific recommendations for future studies, such as investigating ABO blood group associations with COVID-19 in larger, multi-center studies and exploring specific biomarkers in diverse populations. | Thank you, we have edited the Results and Discussion, using this comment and others to summarize the main findings and clearly state the Limitations (see Limitations and Future work section, p. 12-13)  We have added this line to “future work” on page 13: Investigating ABO blood group associations with COVID-19 in larger, multi-center studies may yield other biomarkers of interest. |
| References Section Update with Recent Literature | References have been thoroughly updated as per your suggestions and those of the other reviewers |
| The | Thank you, we summarized the key findings in the conclusion. The limitations of the study are clearly stated in the paragraph immediately before “Conclusion”. |
| Ensure references are updated with the latest literature on ABO blood groups, COVID-19 severity, and relevant biomarkers. Include Contrasting Studies for a Balanced Perspective:  Where possible, add studies with findings that contrast with or complement this study to present a balanced view of the literature. | Thank you we have added the references you and other reviewers suggested. |
| Ensure Consistent Formatting: Confirm that the formatting and accuracy of citations meet journal guidelines | The paper is formatted as per PLOS ONE author guidelines. |
| Reviewer 2 [Dr. Santosh Kumar Sidhwani] |  |
| Introduction: ⎫ Role of markers of endothelial injury and adipocyte activation has not been introduced. These are the primary parameters of study that should be highlighted thoroughly. ⎫ It looks like a discussion, moreover severity, risk factors, markers should by emphasized. | Thank you we have done so, particularly throughout the results section, p. 8-9. |
| Methods: ⎫ “as previously described.” Should be removed from the line no. 167. ⎫  ⎫ What was the criteria used for severity? | Responses:  ⎫ Text has been removed.  ⎫  ⎫ Thank you for your question. We have clarified the clinical guidelines followed for critically ill patients with COVID-19 to the intensive care unit in the methods section as follows:  “*Referral and admission to the ICU was made at the discretion of the attending intensivist in accordance with the Surviving Sepsis Campaign and provincial management guidelines at the time, including all patients requiring mechanical ventilation and once the non-invasively administered oxygen requirements exceed 6 liters/min with a peripheral oximetry saturation of <94% as described*”  [ page 6 ] |
| Inclusion and exclusion criteria not clear. | Thank you, inclusion and exclusion criteria are on page 6:  **Study Design and Participants:** Patients in the present study were enrolled as part of a prospective COVID-19 biomarker study.[42,43] Adult patients admitted to the intensive care unity (ICU) at the Vancouver General Hospital with a diagnosis of pneumonia secondary to SARS-CoV-2 infection between March 30, 2020 and March 31, 2021 were included. Patients were excluded if: COVID-19 was an incidental finding on or during admission (e.g. admitted to ICU primarily for trauma), if COVID-19 was determined to be nosocomial in origin, or if study enrollment took place >10 days after initial ICU admission. |
| Time period of patient recruited is vague. Some where its written from march 2020, to march 2024 and in tables its up to march 2021. | Patients were enrolled and data collected Mar 30, 2020-march 31, 2021. (page 6, lines 168-170)  However, PLOS One requires a statement in the ethics section on when the study data were accessed for analysis – this was Mar 30 2020-Sep 30, 2024 (p 6, lines 161).  Our apologies for any confusion on this, but it is a requirement for the journal. |
| Results: ⎫ Exact p value should be written. ⎫ Comparison of the ABO blood groups with severity should be highlighted before their association with the clinical factors, even though author compared in previous studies but this should be included in supplementary. ⎫.. | Yes, thank you, we have inserted exact p values throughout  We have compared A/AB vs B/O groups throughout |
| ⎫ Steroid, antiviral, immunomodulatory drugs, anti-inflammatory used for no. of days should be highlighted and change in the levels of markers should be highlighted as it is a cohort  Is there any effect of drugs used on adipocyte modification and endothelial injury? ⎫ Except from the markers do author seen any changes in the endothelium or epithelium? ⎫ Levels of the sIL-6R, sgp130, others markers should be compared with the severity and clinical parameters especially comorbid and drugs. These are compared using the regression model so p value should be highlighted in the figures. | Thank you, these are all excellent research questions and excellent research ideas, but beyond the scope of the present study. Nearly all patients in this study were treated with corticosteroids, so we do not have other meaningful comparisons. We did not collect tissue samples to enable comment on endothelial/epithelial changes.  The question of how and if these biomarkers are impacted by factors such as clinical parameters and severity is a very good one – indeed, our group and others have demonstrated that a number of these biomarkers, such as IL-6 or IL-10, are elevated in patients with more severe disease or are associated with respiratory function. For the biomarker analysis, we did not use a regression model, but simply applied a Mann Whitney U test to determine whether there were differences in biomarker levels between participants with blood group A or AB and O or B as that was our main question of interest. A regression model, adjusting for age and sex, was used to determine whether the frequency of specific clinical outcomes ie the need for mechanical ventilation or mortality, as these are well established variables that affect these outcomes. We have added additional text to the statistical methods section to better clarify the approach used. |
| Discussion: Justification of findings should be emphasized with proper references | Yes, thank you, we have updated the references as suggested by reviewer 1 and other reviewers. |
| Reviewer #3: [Anna Lystia Poetranto, DVM., Ph.D] |  |
| 1. “Some 85 studies, including one from our own center in British Columbia, Canada, have shown 86 that blood group A is associated with more severe disease, and blood group O with less 87 severe disease, [6-8] while others have not found an association [9]” , because you mentioned “others have not found” I think it is better to add more references which mentioned it. I saw some unbalance number between references which mentioned an association and no association. | Thank you, we added reference 7 for balance: PMID: [33796389](https://pubmed.ncbi.nlm.nih.gov/33796389/) |
| 2. Related to the hypotheses of “the anti-A theory”, I understand that patients with type O and type B have anti-A antibodies, but the previous paragraph only mentioned about type O and I can’t find the explanation about type B such as what kind of diseases are less or more susceptible to this blood type B. | Thank you, pls see response to reviewer 1 for this issue. |
| 3. The “criteria” for 128 patients, does it mean ARDS criteria in line 188 or ICU criteria or severe COVID-19 criteria? | We have removed the comma such that the statement is clarified that ARDS was diagnosed using the Berlin criteria. Methods, line 188 |
| 4. In line 295 about “Serum cytokines and IL-6 signaling receptors”, you mentioned that no differences in the circulating levels of this cytokine or IL-6 signaling receptors, and you mentioned some references IL-6 signaling receptors which might not explained much about the association of ABO. Don’t you think it is better if you compare with healthy patients just like what your references did? Because your study compared between anti-A antibodies group vs no anti-A antibodies. | This is an excellent question but outside the scope of our study as we did not include healthy controls. |
| 5. If it is possible I want to get clearer explanation and summary of “Serum cytokines and IL-6 signaling receptors” | Thank you for your question. To clarify, serum cytokines refers to IL-6, IL-10, TNFa, and IL-1b , while IL-6 signally receptors refers to sIL-6R and sgp130. |
| 6. You mentioned “Adipsin and leptin are elevated in children with COVID-19” but your sample’s average age is more than 50 years old. Can you summarize or explain whether adult or elder people have the same phenomena or not? | This is an excellent question; to our knowledge there is not a specific study examining this phenomenon in adults/elderly subjects. |
| 7. Since your data were accessed from Mar 30, 2020 to Sep 30, 2021, do you think the SARS-CoV-2 variant influenced the data of patient severity? | This is an excellent consideration. As our participants were enrolled between March 30 2020 to March 31 2021, our cohort will be predominantly composed of patients infected with the wild type SARS-CoV 2 variant. The British Columbia Centre for Disease Control began spot testing ICU patients to determine the virulent strain in late January of 2021, with testing becoming more frequency a month later as BC entered what was considered the third wave in the spring of 2021. Of the 24 participants with genetic data available, 5 were identified as a variant of concern at the time, indicating that the vast majority of patients even in Feb and March of 2021 were infected with the WT strain. This began to shift in April of 2021 as the third wave was driven by variants of concern.  We have added a statement to the discussion to cover this point as follows:  “*Last, we acknowledge the potential influence of SARS-CoV2 variant on our results. While we do not have genetic data on our whole cohort, it is highly likely that the majority of our participants (>80%) were infected with the wild-type strain of SARS CoV 2 given their admission date to the ICU (prior to April 2021) and the timing of the third wave and appearance of the alpha variant of concern in the late spring of 2021 in British Columbia.”* |
| 8. I understand that the sample size indeed is small compared to the duration of your accessed data, what limits the data collection process so that the sample size is small? | Thank you, we included all eligible patients during the study period Mar 2020-March 2021.  Our apologies, there may again be some confusion here – the data for this study were accessed up until Sep 2024, but the actual subjects were only included during that one year period. |
| 9. In line 234-236, you mentioned about the percentage of blood type of your sample, does this percentage numbers also consistent with the percentage of blood type in normal situation in Canada? | This is an excellent question. As we reported in our 2020 paper by Hoiland et al, the estimated prevalence of the blood groups in BC is: 34.1% A, 13.3% B, 4.2% AB, and 48.4% O as compared to our ICU cohort of: 35% A, 23% B, 3.1% AB and 38% O. While it would be interesting to compare the reported percentages, given our relatively small sample size we did not feel we were properly powered to look at blood group frequency as a potential proxy for susceptibility to severe COVID-19 given the additional variables that would need to be incorporated into any model (ie risk of exposure, changes in clinical management between COVID-19 “waves”). Instead, we decide to focus on the blood group based differences of patients admitted to the ICU. |
| 10. From two hypotheses mentioned in line 119-120, in your opinion, which hypotheses is supported by your study data? | As we did not observe any systemic differences in inflammatory of endothelial markers based on ABO but we did confirm increased disease severity in participants with A or AB blood group, our data would support the anti-A theory. |
|  |  |
| Reviewer 4 [Dr. Amr J Halawani] |  |
| Although the small sample size, the article reflects good knowledge and important to the field of the blood groups and COVID-19. Here are some comments to be addressed: | Thank you, please see below for specific responses |
| Line 97: Although it was written in italics, please make it clear that you are mentioned "the ABO gene". | Yes, thank you – the word gene appears after the brackets. |
| Line 180: Define BMI. | Completed (body mass index) |
| Line 191: Define PTT. | Completed (partial thromboplastin time) |
| Line 218: Define IQR. | Completed (interquartile range) |
| Line 304: Please remove the repeated word "elevated". | Completed |
| Overall, very nice work! | Thank you for reviewing the paper! |
|  |  |
| Reviewer #5: |  |
| - Did you consider checking Rhesus factor among your population? | Thank you this is a very good question but beyond the scope of the present study. |
| - If available, Could you elaborate more on low d-dimer levels in discussion part. | Thank you, we have added this sentence to the Discussion: D-dimer in participants with blood group A or AB was lower than those with blood group B or O (1031 vs 1497 ug/L; p=0.03); whether this finding has clinical significance is unclear. [page 12] |
| - For steroids, Was steroids started before or after ICU admission? What was the agent and dosing? | While there were variations in the administration of steroids early in our study (Mar – May 2021), following the publication of the Randomized Evaluation of COVID-19 Therapy (RECOVERY) trial in June 2020, dexamethasone administered at 6 mg po daily up to 10 days or equivalent corticosteroid became the standard of care. Almost all patients were given steroids prior to ICU admission. |
| No tocilizumab alone group? or your guidelines mandates that tocilizumab should be started with of after steroids? | You are correct, we did not have a tocilizumab alone group. In British Columbia, tocilizumab entered widespread use for critically ill COVID-19 patients on Jan 8, 2021, shortly after online publication of the Randomized, Embedded, Multifactorial, Adaptive Platform Trial for Community-Acquired Pneumonia (REMAP-CAP) trial. From January to April, a single dose of 8 mg/kg IV (up to 800 mg), as per the REMAP-CAP and Randomized Evaluation of COVID-19 Therapy (RECOVERY) protocols, was used following the first line response of dexamethasone as described in the response above. |
| - If applicable, it would be bitter to include outcomes on the incidence of thrombosis or bleeding. | Thank you this is an excellent suggestion for future; we did not collect data on clinical clotting or bleeding events for this study. |
